# Multiple Origins or Widespread Gene Flow in Agricultural Fields? Regional Population Genomics of Herbicide Resistance in *Bromus tectorum*

**DOI:** 10.1111/mec.17791

**Published:** 2025-05-08

**Authors:** Victor H. V. Ribeiro, Joseph Gallagher, Carol Mallory‐Smith, Judit Barroso, Caio A. C. G. Brunharo

**Affiliations:** ^1^ Department of Crop and Soil Science Oregon State University Corvallis Oregon USA; ^2^ Research Molecular Biologist, Forage Seed and Cereal Research Unit United States Department of Agriculture Corvallis Oregon USA; ^3^ Department of Plant Science The Pennsylvania State University University Park Pennsylvania USA

**Keywords:** acetolactate synthase, downy brome, gene flow, multiple evolutionary origins, photosystem II

## Abstract

The repeated evolution of herbicide resistance in agriculture provides an unprecedented opportunity to understand how organisms rapidly respond to strong anthropogenic‐driven selection pressure. We recently identified agricultural populations of the grass species *Bromus tectorum* L. with resistance to multiple herbicides. To understand the evolutionary origins and spread of resistance, we investigated the resistance mechanisms to acetolactate synthase (ALS) inhibitors and photosystem II inhibitors, two widely used herbicide modes of action, in 49 *B. tectorum* populations. We assessed the genetic diversity, structure and relatedness in a subset of 21 populations. Resistance to ALS inhibitors was associated with multiple nonsynonymous mutations in *ALS*, the target site gene, despite the relatively small geographic region where populations originated, suggesting ALS inhibitor resistance evolution occurred multiple times in the region. We also found evidence that mechanisms not related to the target site evolved and were common in the populations studied. Resistance to photosystem II inhibitors was confirmed in two populations and was conferred by nonsynonymous mutations in the plastid gene *psbA*. Population genomics analyses suggested that ALS resistance in most populations, at the nucleotide level, spread via gene flow, except for one population where we found evidence that Pro‐197‐His mutations may have evolved in three separate events. Our results suggest that both gene flow via pollen and/or seed dispersal and multiple local evolutionary events were involved in the spread of herbicide‐resistant *B. tectorum* . Our results provide an empirical example of the rapid repeated evolution of a trait under strong anthropogenic selection and elucidate the evolutionary origins of herbicide resistance in a plant species of agricultural importance.

## 1 Introduction

Weeds adapt in response to intensive farming practices (Neve and Caicedo 2022). Over the past two centuries, agricultural practices have rapidly intensified across North America through cropland expansion, monoculture systems and increased chemical inputs (Kreiner, Caballero et al. 2022). Chemical weed control revolutionised weed management by enhancing the levels of weed control while reducing labour input and costs (Swinton and Van Deynze 2017). However, herbicide effectiveness has been challenged by the evolution of herbicide‐resistant weeds (Heap 1997).

The widespread and repeated evolution of herbicide‐resistant weeds is a striking example of weed adaptation to anthropogenic‐driven disturbance. To date, more than 530 unique cases of herbicide‐resistant weeds have been reported (Heap 2025). The evolution of similar adaptive traits in different populations is known as parallel or convergent evolution (Arendt and Reznick 2008). These terms were originally applied to phenotypic traits; however, their use has extended to genes and genetic variants controlling those traits (Elmer and Meyer 2011). For a given species, parallel evolution refers to different populations independently evolving the same genetic changes, while convergent evolution involves different populations evolving similar traits through different genetic changes (Pickersgill 2018). In the context of herbicide resistance, parallel evolution often results in similar resistance phenotypes caused by identical mutations in the same gene, while convergent evolution leads to similar resistance phenotypes through distinct mutations in the same gene. Distinguishing between these forms of repeated evolution requires knowledge of the underlying molecular mechanisms in each population and could be valuable information to identify paths of gene flow in order to target containment efforts, develop molecular tests for rapid resistance detection and improve weed control practices to reduce selection pressure.

In agricultural systems, once a new lethal stress is imposed, adaptive alleles may arise via new mutations, migration of adapted individuals (i.e., gene flow), or the alleles were already present in the ancestor population (i.e., standing genetic variation). There is evidence, however, that these dynamics may be variable depending on the organism and type of stress. Resistance to fungicides, for example, may be conferred by new mutations after the onset of selection pressure (Torriani et al. 2009), while insecticide resistance has been reported to be endowed by new mutations or standing variation (Rose et al. 2011). While *de novo* mutations for herbicide adaptations have not been reported to date, there is evidence that standing genetic variation or gene flow, and a combination of both, are the leading causes of resistance evolution (Cai et al. 2023; Kersten et al. 2023; Kreiner et al. 2019; Lee and Coop 2017). For instance, in *Amaranthus palmeri* and *Alopecurus myosuroides* , resistance seems to arise from standing variation (Kreiner, Latorre et al. 2022; Kersten et al. 2023). Because of agricultural activities, gene flow of herbicide resistance alleles mediated by human activities plays an important role in the spread and introgression of adaptive alleles (Warwick et al. 2008; Brunharo and Tranel 2023; Kreiner, Sandler et al. 2022).

Herbicide resistance mechanisms in weed species can be classified into two types: (1) resistance conferred by mutations in the herbicide target enzyme or gene amplification [target‐site resistance (TSR)] and (2) resistance conferred by mechanisms not involving the target enzyme [nontarget site resistance (NTSR)] (Gaines et al. 2020). Target‐site resistance is primarily determined by monogenic traits (i.e., conferred by large‐effect alleles) (Délye et al. 2013), whereas NTSR can be monogenic or polygenic (i.e., governed by multiple minor‐effect alleles) (Scarabel et al. 2015; Brunharo et al. 2025). Nontarget site resistance can result from physiological and biochemical alterations such as reduced herbicide absorption and translocation, enhanced herbicide metabolism and herbicide vacuolar sequestration (Suzukawa et al. 2021). Although NTSR mechanisms have been extensively characterised, their genetic controls and genetic architecture remain largely unknown. Some examples available in the literature suggest that NTSR may be species vs mechanism‐specific. For instance, there is evidence that NTSR to 4‐hydroxyphenylpyruvate dioxygenase herbicides in *A. tuberculatus* is conferred by a small number of large‐effect loci (Murphy et al. 2021), while NTS glyphosate resistance to this same species involves many small‐effect loci (Kreiner et al. 2021).

*Bromus tectorum* L. (downy brome), a grass species native to the Mediterranean region and Southwest Asia, is a globally widespread weed that has successfully colonised a variety of noncrop disturbed and cultivated habitats (USDA‐ARS 2024). It is a diploid (2*n* = 2*x* = 14), predominantly self‐pollinated, C_3_ winter annual species that only reproduces by seeds (Hulbert 1955; Kao et al. 2008). Despite being predominantly self‐pollinated, outcrossing can occur at low rates (0.27%–1.33%) in this species (Meyer et al. 2013). Research has shown that this species can adapt to local climates, which facilitates its invasion in western North America (Gamba et al. 2024), with potential for expansion to areas with cooler temperatures, higher elevation and drier areas (Boyte et al. 2016; Bradley 2009; Concilio et al. 2013).

In the United States Pacific Northwest, specifically in the northeastern region of Oregon, *B. tectorum* is a problematic weed in grass seed and dryland wheat ( *Triticum aestivum* L.)‐based production systems. It has evolved resistance to several herbicide modes of action, including inhibitors of the acetyl‐coenzyme A carboxylase (ACCase), acetolactate synthase (ALS), photosystem II (PSII), the very‐long‐chain fatty‐acid (VLCFA) inhibitor and 5‐enolpyruvylshikimate‐3‐phosphate (EPSPS) (Ball et al. 2007; Mallory‐Smith et al. 1999; Park et al. 2004; Park and Mallory‐Smith 2004, 2005; Ribeiro et al. 2023; Zuger and Burke 2020). These herbicides work by inhibiting key biochemical pathways, killing the affected plants within days to a few weeks after treatment. Although *B. tectorum* has been a problematic species and resistance has been known since the mid‐1990s (Heap 2025), until recently little was known about the underlying resistance mechanisms.

Our group recently surveyed a small region of Oregon where growers reported control failures with ACCase inhibitors, which is an important mode of action for *B. tectorum* control in fine fescue (*Festuca* L. spp) seed production. We found at least two evolutionary events at the nucleotide level occurred in distinct populations and resulted in ACCase‐inhibitor resistance: nonsynonymous mutations in *ACCase* at codon position 2041 or 2096, both of which are located near the herbicide‐binding domain of the enzyme (Ribeiro et al. 2023). Since those reports, 49 *B. tectorum* populations with various cross‐resistance patterns to ALS inhibitors and multiple resistance to the PSII inhibitor metribuzin were identified in different regions and cropping systems of Oregon (Ribeiro et al. 2024). However, the resistance mechanisms or the genetic relationships among them remain unclear. Inhibitors of ALS were first introduced to the US market in the early 1980s, while PSII inhibitors were introduced much earlier (in the 1970s), and Oregon farmers take advantage of these weed management options because they are safe for the crops they produce (e.g., wheat), while controlling undesirable weeds. The first report of ALS and PSII inhibitors resistance in *B. tectorum* in the region was in 1997 (Mallory‐Smith et al. 1999; Park and Mallory‐Smith 2005).

High levels of phenotypic variations in *B. tectorum* populations are considered the major drivers of *B. tectorum* invasion across different environments (Ashley and Longland 2007; Kao et al. 2008; Merrill et al. 2012). Despite ecological studies regarding the genetic diversity of *B. tectorum* , limited studies have investigated the genetic diversity, structure and relatedness of *B. tectorum* populations from agricultural systems (Lawrence et al. 2017). Its ability to adapt to herbicides, persist across growing seasons and compete with cereal and grass seed crops has made it particularly challenging to manage (Ostlie and Howatt 2013; Stahlman and Miller 1990). Understanding the genetic relationship among populations from different agricultural regions and cropping systems could help us understand whether resistant populations are evolving independently or their widespread infestation is due to natural or human‐facilitated gene flow. In turn, dissecting the source of adaptive alleles could help us design management strategies to reduce herbicide selection pressure locally and advise policymakers and growers on measures to limit allele movement. For example, genetic markers could be developed to quickly identify whether a farm is infested by an herbicide‐resistant population, which would allow farmers to use this information to choose the proper herbicide prior to treatment. In this study, we hypothesised that the herbicide‐resistant *B. tectorum* populations identified in the region are the result of a few evolutionary events that spread in the region facilitated by human activities. This hypothesis stems from the fact that agricultural activities (e.g., seed trade and movement of farm implements) facilitate the dispersal of herbicide resistance alleles in this region. Our hypothesis will be tested by elucidating the relatedness among populations using a restriction enzyme‐associated DNA sequencing (RAD‐seq) approach, as well as the genetic variants associated with resistance to key herbicides in this cropping system using Sanger sequencing. If our hypothesis is correct, we would observe identical mutations in the herbicide target‐site across multiple *B. tectorum* populations, and different populations would share the same genetic background. Alternatively, if resistant populations harbour distinct resistance‐endowing mutations and ancestries, then convergent evolution is the most likely explanation for the widespread resistance in *B. tectorum* . In this scenario, we would observe distinct resistance‐endowing mutations in the herbicide target‐site and a more heterogeneous distribution of genetic ancestries. Therefore, this study aimed to (1) investigate whether there are nucleotide polymorphisms in *B. tectorum* associated with resistance to key herbicides and (2) dissect the genetic diversity, structure and relatedness of herbicide‐resistant *B. tectorum* populations from winter wheat and fine fescue fields.

## 2 Material and Methods

### 2.1 Plant Material for Phenotyping and Gene Sequencing Analyses

Forty‐nine *B. tectorum* populations collected from winter wheat fields in northeastern Oregon were used in this study. Populations were collected in 2021 and 2022 as part of a survey for herbicide resistance in *B. tectorum* (Ribeiro et al. 2024). The samples originated from 49 fields managed by 28 growers, who primarily relied on ALS inhibitors (76% use) and metribuzin (16% use) to control downy brome.

### 2.2 Phenotyping

Sulfosulfuron and metribuzin were chosen as herbicides representative of ALS and PSII inhibitors, respectively, to phenotype plants as resistant or susceptible, because of their widespread use in winter cereal production systems in the region. Approximately 30 seeds of each population were germinated in acrylic square boxes (10 cm width × 10 cm length × 2.5 cm height; 156C container, Hoffman Manufacturing Inc., Corvallis, OR) in a growth chamber with continuous light at 15°C. Seedlings (approximately 5 cm of shoot length) were transplanted into four‐celled trays (6 cm width × 6 cm length × 5.7 cm depth; Grower's Nursery Supply Inc., Salem, OR) filled with a commercial potting mix (SS #4 PC RSi, Sun Gro Horticulture, Agawam, MA). Plants were grown in a greenhouse at Oregon State University, Corvallis, OR, (44.56° N, 123.28° W) with 24/15°C day/night, supplemented with 400 W high‐pressure sodium light bulbs (350 μmol m^−2^ s^−1^) to ensure a 12 h photoperiod. Four to five *B. tectorum* plants of each population were treated at the recommended field rate of sulfosulfuron (Outrider; 35 g ai ha^−1^) or metribuzin (TriCor DF; 420 g ai ha^−1^) when plants were at the two‐ to three‐leaf stage. The recommended herbicide field rates were previously used in the study by Ribeiro et al. (2024) to discriminate between resistant and susceptible individuals. Treatments were applied using a research sprayer (DeVries Manufacturing, Generation III, Hollandale, MN) delivering 140 L ha^−1^ spray volume through a single TP8003E nozzle (TeeJet Technologies, Wheaton, IL, USA). *Bromus tectorum* plants were visually assessed as dead (completely necrotic plants; assessed value of 0) or alive (green tissue remaining and evidence of regrowth; assessed value of 1) 21 days after treatment.

### 2.3 DNA Extraction and *ALS* and *psbA* Gene Sequencing

Young leaf tissue (approximately 50 mg) was collected from four to five plants of each of the 49 field‐collected *B. tectorum* populations before herbicide treatment. Samples were submitted to the Core Facilities of the Center for Quantitative Life Sciences of Oregon State University, Corvallis, Oregon, for DNA extraction. Herbicide resistance status of sequenced plants was assessed 21 days after treatments, as previously described. Data on the presence or absence of known resistance‐endowing mutations in the herbicide target sites were generated.

We used three primer sets (Table S1) to amplify the five conserved domains (A, B, C, D and E) of the *ALS* gene, spanning the eight amino acid positions (Ala‐122, Pro‐197, Ala‐205, Asp‐376, Arg‐377, Trp‐574, Ser‐653 and Gly‐654) responsible for resistance to ALS inhibitors (Tranel et al. 2025), to test whether SNPs were involved in the resistance mechanism. These positions have been extensively characterised and reported in at least 180 weed populations (Tranel et al. 2025). Amino acid substitutions at these positions play a crucial role in herbicide binding and inhibition of ALS (Garcia et al. 2017). Although other SNPs could contribute to herbicide resistance, such cases are unlikely. Polymerase chain reactions (PCR) were performed in a C‐1000 Touch Thermal Cycler (Bio‐Rad Laboratories, Oslo, Norway). PCR (30 μL total volume) contained 6 μL of 5 × Phusion HF Buffer (New England Biolabs, Ipswich, MA, USA), 0.6 μL of 10 mM dNTPs (Thermo Fisher Scientific, Waltham, MA, USA), 1.5 μL of each forward and reverse primer (10 μM), 6 μL of gDNA (10 ng μL^−1^), 0.15 μL of Phusion High‐Fidelity DNA Polymerase (Thermo Fisher Scientific) and 14.25 μL of nuclease‐free water. A 1271‐bp fragment covering the known codon positions Ala‐122, Pro‐197 and Ala‐205 was amplified using the following conditions: 60 s denaturing at 98°C, 40 cycles of 10 s elongation at 98°C, 30 s of annealing at 68°C, 60 s of elongation at 72°C and a 10 min final extension at 72°C. For the 762‐bp fragment covering the known codon positions Asp‐376 and Arg‐377, the annealing temperature used was 66°C. For the 556‐bp fragment covering the known codon positions Trp‐574, Ser‐653 and Gly‐654, the annealing temperature was 62°C. PCR products were run on a 1% agarose gel stained with GelRed Nucleic Acid Gel Stain (Biotium, Fremont, CA, USA) to visualise amplicon size. Amplicons were sent to Eurofins Genomics (Louisville, KY; eurofinsgenomics.com) for sample purification and Sanger sequencing.

We aligned the resulting sequences to a reference *B. tectorum ALS* gene sequence (AF488771.1) obtained from the GenBank database of the National Center for Biotechnology Information (NCBI) using Geneious Prime software (version 2022.2) to visually inspect for mutations at the eight positions in the *ALS* gene known to confer resistance to ALS inhibitors. We amplified a 1095‐bp fragment of the conserved chloroplast *psbA* gene containing the seven known codon positions (Leu‐218, Val‐219, Ala‐251, Phe‐255, Ser‐264, Asn‐266 and Phe‐274) responsible for conferring resistance to PSII‐inhibiting herbicides (Lu et al. 2019; Powles and Yu 2010; Thiel and Varrelmann 2014), using the forward (5′‐ACCATGACTGCAATTTTAGAG‐3′) and reverse (5′‐AAAATTCTTATATGTTAGCA‐3′) primer sequences previously described by Park and Mallory‐Smith (2005) to test the hypothesis that resistance to metribuzin in *B. tectorum* populations was due to a target site‐based resistance mechanism. PCR reactions contained 30 μL total volume as previously described for *ALS* gene sequencing. The *psbA* gene was amplified following the same conditions as for *ALS*, except the annealing temperature was 52°C. Amplicon size was checked, and samples were sent to Eurofins Genomics for purification and Sanger sequencing. We aligned the resulting sequences to a reference *B. tectorum psbA* gene sequence (AY744774.1) obtained from GenBank to inspect for mutations at the seven positions in the *psbA* gene known to confer resistance to PSII inhibitors.

### 2.4 Plant Material for Population Genomics Analysis

We used a subset of 21 *B. tectorum* populations for the population genomics investigations (Table S2). Nineteen of the 49 *B. tectorum* populations used for phenotyping were collected from winter wheat fields in Gilliam, Morrow, Sherman, Umatilla and Wasco Counties, Oregon and confirmed as ALS or PSII inhibitor‐resistant (Table S2). Two *B. tectorum* populations collected from fine fescue fields in Union County, Oregon, previously confirmed as ACCase‐resistant in a previous study, were included (Ribeiro et al. 2023). We selected the populations based on their resistance patterns and the geographic distance between collection sites. We hypothesised that long‐distance gene flow of *B. tectorum* may occur among crop production systems, potentially mediated by agricultural trade and movement of farm implements.

### 2.5 Population Genomics of *B. tectorum* Populations

To genotype the *B. tectorum* populations, we sequenced four to five individuals per population using a RAD‐seq approach (Elshire et al. 2011). A closely related species, *Bromus diandrus* Roth, was used as an outgroup (population WAS6). We utilised DNA from the same extraction used for *ALS* and *psbA* sequencing analyses for the RAD‐seq experiment. A total of 95 libraries were generated by sample digestion with the restriction enzyme *PstI*, tagged with individual barcodes and paired‐end sequenced (2 × 150 bp) in an Illumina NovaSeq 6000 (Floragenex, Eugene, Oregon). A total of 542 M reads were generated, with an average of 7,957,966 ± 695,762 reads per sample. We used *Stacks* (v2.64; Rochette et al. 2019) pipeline for SNP identification. First, we demultiplexed samples using the *process_radtags* program in paired‐end mode and used *PstI* as the restriction enzyme in the DNA library step. Second, we aligned the sample reads to the *B. tectorum* reference genome (Revolinski et al. 2023) using *bwa mem* (v0.7.17; Li and Durbin 2009) and sorted using *SAMtools* (v1.6; Li et al. 2009). Finally, we executed the *ref_map* program with default parameters to generate loci to be genotyped. The last step was to run the *populations* program that applies a population genetics framework to the aligned *bam* files. We recognise that aligning the *B. diandrus* DNA sequences to the *B. tectorum* genome could impact downstream analysis by reducing the number of successfully mapped reads, mapping quality and number and depth of SNPs identified (Bohling 2020). To overcome these limitations, we only included SNPs that were present in at least 90% of the samples within a population (i.e., *‐r* 0.9) and present in all populations (i.e., ‐*p* 22). We generated bootstrap *p* values for population summary statistics using the *populations* module of Stacks with the *–bootstrap* flag, and the *–smooth* flag to report summary statistics values based on sliding‐windows (default of 450 kbp). The SNP identification approach resulted in 170,641 SNPs, with a mean depth of 10 ± 6.1 × (± SE). The scripts for these analyses are available in https://github.com/caiobrunharo/Bromus_tectorum_Oregon/.

The first step to better understand the relationship among populations was to visualise the SNP dataset with a principal component analysis (PCA), which is a model‐free data summary statistic to provide an overview of the population structure (McVean 2009). If a set of populations exhibits the same ALS resistance‐endowing mutations and cluster together in the PCA, it would suggest a single evolutionary origin explaining the resistance phenotype. Alternatively, if individuals with the same mutation cluster by population, with those populations positioned apart from each other in the PCA, it would suggest that the mutation arose independently in each population, consistent with multiple evolutionary origins. Next, we used the software ADMIXTURE (v1.3.0; Alexander et al. 2009) to dissect the patterns of ancestry among the populations studied. We sorted the genotype file from *Stacks* using the *run_pipeline* module of TASSEL (v5.2.40; Bradbury et al. 2007) and converted it to a binary file using *plink* (v1.90b6.21; Purcell et al. 2007). We then ran ADMIXTURE with *K* values varying from 1 to 15 and visualised it using *PONG* (v1.5; Behr et al. 2016), following the commands provided in Liu et al. (2020). We performed a cross‐validation analysis within ADMIXTURE to help identify the most likely true value of *K* (Liu et al. 2020). The cross‐validation step relies on the identifications of *K* values in which the model has the greatest predictive accuracy (Alexander and Lange 2011). If the resistance‐endowing variant evolved once and then spread in the region, we would see shared ancestry in the ADMIXTURE plots. Conversely, if resistant populations with the same resistance‐endowing mutations have distinct ancestries, then it would support the hypothesis of independent evolution. Finally, we obtained the population genomics summary statistics expected heterozygosity (H_E_), nucleotide diversity (π) and inbreeding coefficient (F_IS_) using *Stacks*. The H_E_ and π datasets were generated to support the hypothesis that more diverse populations could have more potential to evolve resistance, or whether recent selection pressure from herbicides could have reduced the genetic diversity and potential for evolution. The F_IS_ data could indicate whether populations have experienced admixture, supporting the hypothesis of gene flow of herbicide resistance in the region. We performed an isolation‐by‐distance test to understand whether the geographical isolation of populations contributed to their distinct genotypes. By plotting the genetic distances over the geographic distances, we could infer whether there were patterns in their correlations and further support the hypothesis of widespread gene flow of resistance. We performed a Mantel test using the *adegenet* R package (Jombart 2008) by correlating the pairwise genetic distance using the Euclidean method (obtained with the *dist.genpop* function from *adegenet*) among individuals with their geographical distances using scripts available from https://github.com/thibautjombart/adegenet. Lastly, we constructed a Neighbour‐joining tree using the *ggtree* (Xu et al. 2022) and *treeio* (Wang et al. 2019) R packages by plotting the pairwise genetic distances (obtained with the *adegenet* package) for tree estimation with the *nj* function from the *ape* package (Paradis et al. 2004). If populations with resistance‐endowing mutations are spread across different clades in the phylogenetic tree, it could indicate multiple evolutionary events of resistance evolution.

## 3 Results

### 3.1 Phenotyping and *ALS* and *psbA* Gene Sequencing Analyses

**FIGURE 1:**
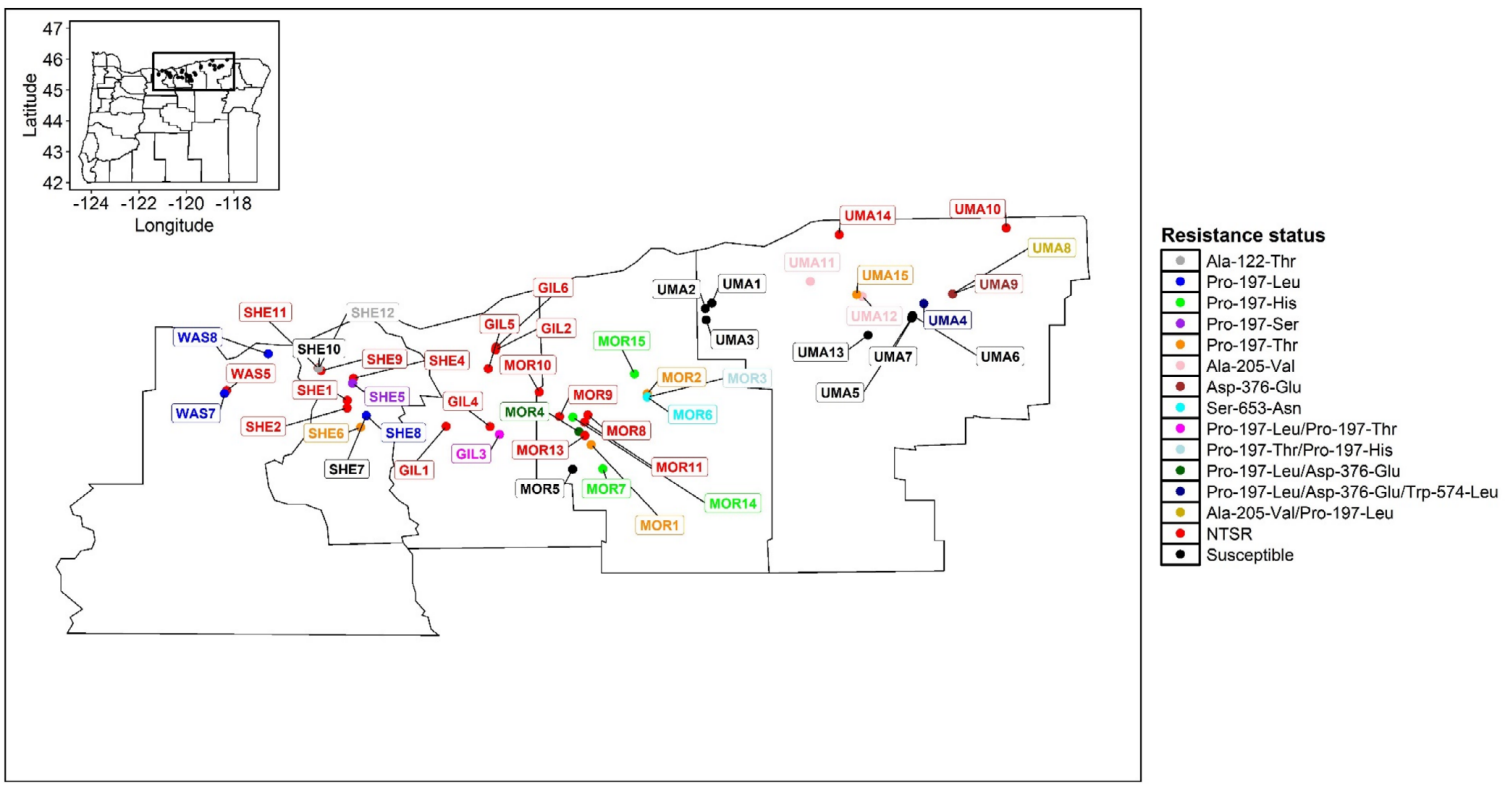
Distribution of ALS‐resistant *Bromus tectorum* populations across Oregon. Coloured dots indicate the resistance status, including target‐site resistance (TSR) mutations, nontarget‐site resistance (NTSR) and susceptibility.

*B. tectorum* populations displayed resistance to both ALS‐ and PSII‐inhibiting herbicides (Figure 1; Table S3). Therefore, we sequenced the *ALS* and *psbA* genes to determine if amino acid substitutions known to confer ALS and PSII inhibitor resistance, respectively, were present in these genes. Twenty‐one of the 49 *B. tectorum* populations had at least one individual with a resistance‐endowing nonsynonymous mutation in the *ALS* gene (Figure 1; Table S3). Mutations were found in six different positions in the *ALS* gene: Ala‐122, Pro‐197, Ala‐205, Asp‐376, Trp‐574 and Ser‐653, including four distinct amino acid substitutions at Pro‐197 and combinations thereof. Sixteen populations had plants with a single mutation, four populations had two different mutations and one population had three different mutations. No individual with more than one nonsynonymous mutation was identified. Eighteen populations survived sulfosulfuron treatment but did not have a mutation in any of the eight known resistance‐endowing amino acid positions of the *ALS* gene, suggesting that NTSR mechanisms may be involved. Ten populations were susceptible to sulfosulfuron and had wild‐type alleles at the eight amino acid positions investigated in the *ALS* gene.

For the *psbA* gene sequence analysis, a mutation in the Ser‐264 position was identified in two *B. tectorum* populations (Table S3). Both populations were multiple herbicide‐resistant (i.e., resistant to more than one herbicide mode of action), given they had the Ser‐264 mutation in the *psbA* and the Pro‐197‐Leu and/or Pro‐197‐Thr mutations in the *ALS*.

### 3.2 Population Genomics of *B. tectorum* Populations

We investigated the genetic diversity, structure and relatedness of a subset of 19 populations collected from wheat fields and two populations from fine fescue fields, using *B. diandrus* as an outgroup to understand the evolution and spread of herbicide resistance. We observed a wide variation in the population genomics statistics in *B. tectorum* (Table 1). The H_E_ and π estimates had similar trends, ranging from 0.0029 to 0.042 and 0.0032 to 0.046, respectively, while the outgroup ( *B. diandrus* ; WAS6) had greater H_E_ (0.13) and π (0.16). The F_IS_ estimates ranged from −0.00058 to 0.086, and the outgroup was −0.02504. These results suggest a wide variation in the population genomics estimates, with a 14.5‐, 14.4‐ and 956‐fold change in H_E_, π and F_IS_ respectively.

**TABLE 1:** Summary of population genetics estimates.

Population	*H*_*E*_	*π*	*F*_*IS*_
GIL3	0.032 ± 0.00037	0.037 ± 0.00043	0.064 ± 0.0017
GIL5	0.034 ± 0.00043	0.040 ± 0.00049	0.056 ± 0.00080
MOR1	0.0034 ± 0.000098	0.0038 ± 0.000098	−0.00058 ± 0.00020
MOR3	0.0042 ± 0.000098	0.0047 ± 0.00012	−0.00055 ± 0.00027
MOR5	0.033 ± 0.00037	0.038 ± 0.00043	0.066 ± 0.0010
MOR10	0.018 ± 0.00026	0.020 ± 0.00027	0.040 ± 0.00045
MOR14	0.032 ± 0.00039	0.037 ± 0.00045	0.059 ± 0.00055
MOR15	0.0072 ± 0.00014	0.0082 ± 0.00018	−0.00047 ± 0.00072
SHE4	0.018 ± 0.00027	0.021 ± 0.00031	0.032 ± 0.00057
SHE6	0.038 ± 0.00041	0.043 ± 0.00047	0.075 ± 0.0010
SHE7	0.020 ± 0.00027	0.023 ± 0.00031	0.037 ± 0.00078
SHE9	0.015 ± 0.00026	0.018 ± 0.00027	0.025 ± 0.00088
UMA2	0.042 ± 0.00043	0.046 ± 0.00047	0.086 ± 0.00057
UMA7	0.0048 ± 0.00012	0.0055 ± 0.00014	0.00056 ± 0.0016
UMA8	0.016 ± 0.00023	0.018 ± 0.00027	0.022 ± 0.00076
UMA14	0.031 ± 0.00037	0.036 ± 0.00043	0.053 ± 0.0014
UMA15	0.0029 ± 0.000098	0.0032 ± 0.000098	−0.00038 ± 0.0015
UDB1	0.0049 ± 0.00012	0.0054 ± 0.00014	−0.000090 ± 0.0017
UDB9	0.0055 ± 0.00012	0.0062 ± 0.00014	−0.00055 ± 0.00053
WAS5	0.0066 ± 0.00016	0.0076 ± 0.00018	−0.00017 ± 0.00086
WAS8	0.0048 ± 0.00012	0.0053 ± 0.00012	−0.00035 ± 0.00080
WAS6 (outgroup)^a^	0.13 ± 0.00071	0.16 ± 0.00084	−0.025 ± 0.0

Abbreviations: *π*, nucleotide diversity; *F*_*IS*_, inbreeding coefficient; *H*_*E*_, expected heterozygosity over polymorphic sites; means ±95% confidence intervals.

^a^ WAS6 is *Bromus diandrus* and was used as an outgroup.

**FIGURE 2:**
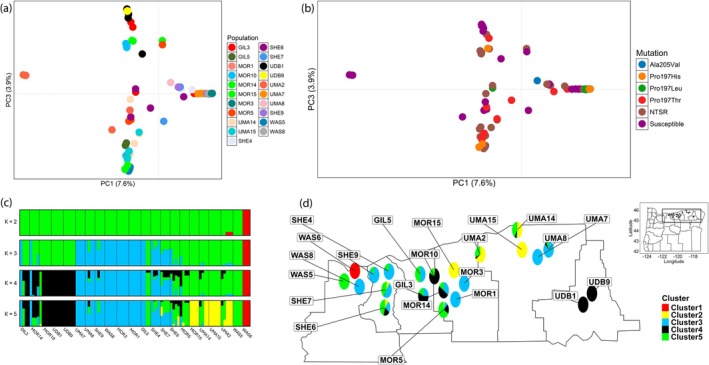
(a and b) Principal component analysis of *Bromus tectorum* populations. (a) underscoring the separation of populations. (b) underscoring the separation based on amino acid substitution in the *ALS*. NTSR represents individuals phenotyped as resistant but without mutations in the *ALS*. (c and d) Admixture analysis of genetic clusters in *B. tectorum* populations. (c) Probabilities of assignment of individuals at *K* = 2–5. WAS6 population is the outgroup, *B. diandrus* . (d) Geographic distribution of genetic clusters (*K* = 5) in *B. tectorum* populations in Oregon. *B. tectorum* populations are represented by four distinct clusters (yellow, blue, black and green). WAS6 population is the outgroup, *B. diandrus* (red cluster).

We performed a PCA to understand the genetic relationships among the *B. tectorum* populations. The PCA plot, which includes the outgroup *B. diandrus* (WAS6), showed a clear distinction between the outgroup and the main cluster of *B. tectorum* populations along both PC1 (31.5%) and PC2 (10.4%) (Figure S1a). This clear separation, as expected, indicates substantial genetic differentiation between *B. diandrus* and *B. tectorum* . The removal of the outgroup from the analysis allowed for a clearer view of the genetic variation among *B. tectorum* populations, as evidenced by a continuous distribution of individuals along both axes (Figure 2a,S1b). The genetic relationships were further dissected by identifying individuals based on their specific mutation in the *ALS* gene (Figure 2b) or resistance status (Figure S1c). Convergent evolution was observed at both gene and amino acid levels. At the gene level, different mutations in *ALS* appeared across multiple regions of the PCA (Figure 2b). At the amino acid level, we observed the wild‐type proline at position 197 replaced by histidine, leucine or threonine, indicating convergent evolution in distinct populations that gave rise to the same resistant phenotype. In contrast, the repeated evolution of the same specific mutation, Pro‐197‐His, in three genetically distinct populations (MOR3, MOR14 and MOR15), which are separated in the PCA (Figure S1d), provides evidence of parallel evolution in our system. However, additional experiments would be necessary to draw more definite conclusions about whether Pro‐197‐His evolved independently multiple times. For instance, the genomic regions surrounding *ALS* could be sequenced using long‐read technology to achieve sufficient resolution for haplotype identification, as performed by Kersten et al. (2023).

We performed an ADMIXTURE analysis to dissect the genetic structure of the populations, and the results were largely consistent with the PCA. At *K* = 2, where *K* represents the number of genetic clusters in the analysis, there was a clear distinction between the outgroup *B. diandrus* (WAS6; red) and the *B. tectorum* populations (green) (Figure 2c). As *K* increased, the analysis revealed finer‐scale population structure, introducing additional clusters (blue, black and yellow) that further partitioned genetic ancestry among the individuals. Cross‐validation analysis (Figure S2) identified *K* = 5 as the most likely number of genetic clusters; therefore, we present plots for *K* = 2–5. At *K* = 5, clearer genetic relationships were observed among several sets of populations with uniform genetic structure: UDB1 and UDB9 (black), UMA7, WAS8, MOR3 and MOR1 (blue), MOR15 and UMA15 (yellow) and WAS5 alone (green) (Figure 2c). Gene flow or admixture was evident in some populations, indicated by the mix of individuals from different ancestries within populations (GIL3, MOR14, MOR10, UMA8, SHE9, GIL5, SHE4, SHE7, SHE6, MOR5, UMA14 and UMA2). In particular, gene flow mediated by seed movement is evident by the presence of individuals assigned to an ancestry distinct from the rest of the individuals in that population, such as an individual assigned to the blue ancestry in GIL3 or individuals with green ancestry in GIL5. In contrast, admixture events were observed in populations where individuals showed mixed ancestry. For example, in SHE9, all individuals were assigned to the blue ancestry except one with a proportion of green, while in UMA8, most individuals were blue with one individual showing proportions of green and black. Similarly, all individuals from SHE7 had some degree of blue and green ancestry, and those from SHE6 had proportions of green, black, blue and yellow ancestries (Figure 2c). These patterns suggest a combination of seed‐mediated or pollen‐mediated gene flow contributing to admixture in these populations. These findings align with the PCA results (Figure 2a), where we observed the ADMIXTURE black cluster populations positioned at the top of the PCA space, the yellow at the bottom, the blue on the right and admixed individuals distributed in the centre. Phylogenetic clustering also largely corroborated the ADMIXTURE analysis (Figure S3). For instance, the blue cluster in the ADMIXTURE analysis was also identified as a large clade on the phylogenetic tree (blue arrow; Figure S3). The close agreement between these three independent analyses further supports the evidence that *K* = 5 is appropriate for a more in‐depth discussion of our findings.

Geographically close populations may be more likely to undergo gene flow and share ALS‐inhibiting herbicide resistance mutations. However, genetic clusters derived from the ADMIXTURE analysis did not show a clear structure when mapped (Figure 2d). Indeed, the hypothesis of isolation‐by‐distance was rejected (Mantel test, *p* = 0.15), indicating that geographically closer populations were not necessarily more genetically similar to those farther apart (Figure S4). This lack of isolation‐by‐distance could be explained by a combination of gene flow over both short and long distances and the effects of admixture, which supports our ADMIXTURE data.

## 4 Discussion

Our findings suggest multiple events of convergent evolution, with distinct *ALS* mutations leading to the same herbicide resistance phenotype across populations. In addition to identifying several mutations in *ALS*, the results revealed extensive admixture among genetic clusters across different populations, indicating gene flow in this predominantly self‐pollinated plant species. We found distinct mutations in *ALS* resulting in resistance to sulfosulfuron in several *B. tectorum* populations. Some *B. tectorum* populations resistant to sulfosulfuron did not have *ALS* mutations, suggesting the presence of NTSR mechanisms. Resistance to the PSII inhibitor metribuzin was identified in two *B. tectorum* populations and is associated with a target site mutation in the *psbA* gene. The ability of a weed population to adapt to management practices, including herbicide applications, depends on the presence of the necessary genetic variation (Kreiner et al. 2018). Gene flow among populations has been shown to contribute to long‐distance dispersal of herbicide resistance, facilitated by agricultural activities and could be involved in facilitating the evolution of herbicide resistance in *B. tectorum* (Gonçalves Netto et al. 2021; Brunharo and Streisfeld 2022).

Depending on the mutated amino acid position, specific amino acid substitutions, and the number of resistance alleles in the *ALS* gene, different resistance levels and patterns have been reported in several weed species (Tranel et al. 2025; Yu and Powles 2014). In this study, nine different *ALS* mutations were responsible for resistance to the ALS inhibitor sulfosulfuron in 21 of the 49 *B. tectorum* populations: Ala‐122‐Thr (GCG‐ACG), Pro‐197‐His (CCC‐CAC), Pro‐197‐Thr (CCC‐ACC), Pro‐197‐Leu (CCC‐CTC), Pro‐197‐Ser (CCC‐TCC), Ala‐205‐Val (GCG‐GTG), Asp‐376‐Glu (GAC‐GAG), Trp‐574‐Leu (TGG‐TTG) and Ser‐653‐Asn (AGC‐AAC). Although exceptions exist, generally mutations in ALS at Pro‐197 confer resistance to sulfonylurea and triazolopyrimidine herbicides, mutations at Ala‐122, Ala‐205, Ser‐653 or Gly‐654 confer resistance to imidazolinone herbicides, and mutations at Asp‐376, Arg‐377 or Trp‐574 confer broad‐spectrum resistance across all ALS‐inhibiting chemical families (Tranel et al. 2025). Although the *B. tectorum* populations were only screened for resistance to sulfosulfuron in this study, multiple cross‐resistance patterns were identified in these populations previously when tested against other ALS‐inhibiting herbicides (Ribeiro et al. 2024). The presence of NTSR cannot be ruled out as 18 populations survived sulfosulfuron treatment but did not carry a resistance‐endowing mutation in *ALS*. Previous research identified a *B. tectorum* population where ALS inhibitor resistance was mediated by enhanced metabolism via cytochrome P450 monooxygenase enzyme (Park et al. 2004).

The *psbA* sequence analysis indicated that the Ser‐264‐Gly (AGT‐GGT) mutation detected in two *B. tectorum* populations was the molecular basis of resistance to the PSII inhibitor metribuzin. These populations also exhibited multiple resistance to the ALS inhibitor sulfosulfuron and had a mutation in the *ALS* gene. The PSII resistance‐endowing Ser‐264‐Gly mutation has been previously identified as the mechanism of resistance to metribuzin in a *B. tectorum* population from Oregon, USA (Park and Mallory‐Smith 2005). That population was also ALS inhibitor‐resistant due to a P450‐mediated enhanced metabolism, an NTSR mechanism (Park et al. 2004).

The genetic diversity estimated (H_E_ = 0.0029–0.042 and π = 0.0032–0.046) among *B. tectorum* populations in our study was generally low and is within the lower range of previous estimates for *B. tectorum* populations (H_E_ = 0.021–0.667) collected from small grain production fields in the Pacific Northwest (Lawrence et al. 2017). Comparable low levels of heterozygosity have also been reported for other self‐pollinated species including *Conyza bonariensis* (H_E_ = 0.00–0.35) (Okada et al. 2014), *C. canadensis* (H_E_ = 0.00–0.45) (Okada et al. 2013) and *Echinochloa oryzoides* (H_E_ = 0.089–0.693) (Osuna et al. 2011), although the upper values in these studies were substantially higher than those observed in our study. The differences in H_E_ between our study and previous studies can be explained by both the sequencing techniques and the number of individuals used per population. We sequenced four to five individuals per population using RAD‐seq, which samples a small fraction of the genome (~0.05%) but generates thousands of SNPs across many loci. In contrast, Okada et al. (2013, 2014) and Osuna et al. (2011) used microsatellite analysis and sequenced 30 and 20 individuals per population, respectively, targeting fewer loci but with more comprehensive representation within populations. Lawrence et al. (2017) sequenced only one individual per population using genotype‐by‐sequencing approach, which captures broader genomic variation but may miss within‐population diversity. Therefore, both the number of individuals per population and the genomic coverage of each genotyping method likely contributed to the differences observed in genetic diversity estimates across studies. The H_E_ had a relationship with the ADMIXTURE analysis, in that the populations with the greatest levels of H_E_ (e.g., UMA2, SHE6) were assigned to multiple population clusters, suggesting much of the genetic diversity is driven by the admixture with individuals from different genetic backgrounds, rather than average diversity within individuals.

Recently, Gamba et al. (2024) studied the role of local adaptation in the range expansion of *B. tectorum* in Western North America and found that nucleotide diversity was much greater in populations from the Western United States (π = 0.0016 ± 0.0000045) than from the Eastern United States (π = 0.0009 ± 0.0000045). *B. tectorum* is considered one of the most troublesome invasive species in natural areas in the Western United States, and the authors suggested that greater genetic diversity in populations from the region could contribute to the colonisation success. Interestingly, all *B. tectorum* populations that we studied had greater π than those reported by Gamba et al. (2024), suggesting mechanisms that increase genetic diversity (e.g., multiple introductions, gene flow, frequent disturbances in agroecosystems) could contribute to herbicide resistance evolution. The F_IS_ estimates, −0.00058 to 0.086, indicated that *B. tectorum* populations exhibited low to moderate levels of inbreeding. Thirteen of the 21 populations exhibited positive F_IS_ values, whereas the populations with negative F_IS_ values showed only marginal deviations (ranging from −0.00009 to −0.00058), indicating limited inbreeding. Most F_IS_ values were close to zero, indicating minimal inbreeding across populations, where six of the 21 *B. tectorum* populations had F_IS_ of zero (i.e., confidence intervals contained zero; Table 1) with some populations showing slight deviations towards either heterozygote excess or deficiency. The negative F_IS_ values observed in certain populations suggest occasional outcrossing, which may facilitate gene flow between populations. In contrast, positive F_IS_ values reflect moderate inbreeding, likely associated with higher rates of self‐pollination or limited gene flow. These variations in F_IS_ could be influenced by a combination of factors, including local environmental conditions, selective pressures, and the balance between inbreeding and outcrossing, which collectively shape the genetic structure of *B. tectorum* populations.

Principal component analysis, ADMIXTURE and phylogenetic analysis suggested that the spread of herbicide resistance could be explained by both gene flow among populations, as well as independent evolution of the same mutations in different populations. The ADMIXTURE analysis revealed clear population structure with substantial admixture among genetic clusters (Figure 2c). The presence of admixed individuals strongly indicates ongoing gene flow between these clusters. Gene flow refers to the transfer of genetic material between different populations of the same species or between species, facilitated by processes such as migration, reproduction or hybridisation (Slatkin 1985). This exchange of genetic material can introduce new variations into a population, potentially altering its genetic structure and influencing its evolutionary path (Mallory‐Smith et al. 2015). In plants, gene flow can occur through the dispersal of pollen, seeds, and/or vegetative propagules (Jhala et al. 2021; Mallory‐Smith and Zapiola 2008). Weed seed dispersal, in particular, can spread herbicide resistance alleles on a larger scale compared to pollen flow (Beckie et al. 2019; Gonçalves Netto et al. 2025). With our dataset, we are unable to determine whether the beneficial alleles arose in the population via standing variation or *de novo* mutations, because a more detailed characterisation of the *ALS* locus and its surrounding region would be required (Lee and Coop 2017).

In this study, we found evidence of long‐distance gene flow between *B. tectorum* populations from distinct regions (Union vs. Gilliam, Morrow, Sherman, Umatilla and Wasco Counties) and cropping systems (fine fescue vs. winter wheat). Populations collected from fine fescue fields in Union County (UDB1 and UDB9) had a similar genetic structure represented by black, which is present in admixed populations (GIL3, MOR5, MOR10, MOR14, SHE4, SHE6, SHE7, UMA2, UMA8 and UMA14) collected from winter wheat fields across other counties (Gilliam, Morrow, Sherman, Umatilla and Wasco). These findings are consistent with the Mantel test, which found no significant correlation between genetic distance and geographic distance, suggesting that the spread of resistance alleles is not constrained by geographic proximity. Thus, processes such as gene flow via pollen and/or seed dispersal, strong selection pressure and multiple evolutionary events likely drive the spread of herbicide resistance across the landscape. Similar mechanisms of resistance spread have been observed in several highly self‐pollinated weed species (Lu et al. 2007; Merriam et al. 2023; Okada et al. 2013, 2014; Osuna et al. 2011).

Although widespread gene flow is the primary means of herbicide resistance dispersal in the region, we also found strong evidence of parallel evolution (i.e., independent evolution of the same resistance‐endowing mutation in different population) for the mutation Pro‐197‐His, for three reasons. First, we found that individuals from populations MOR3, MOR14 and MOR15, with the Pro‐197‐His mutation, fall on different sections of the PCA plot (Figure S1d). Second, we observed that MOR3, MOR14 and MOR15 are assigned to different ancestries in the ADMIXTURE plot (MOR3 is blue, MOR14 is primarily black and MOR15 is yellow; Figure 1c). Third, our phylogenetic analysis placed these three populations in three distinct clades. Taken together, results from PCA, ADMIXTURE and the phylogenetic tree suggest potential independent evolutionary events for Pro‐197‐His. Nevertheless, more research is needed to determine whether these are indeed independent evolutionary events.

Our PCA analyses also suggested that NTSR (i.e., individuals that survived herbicide treatment but did not have mutations in the *ALS*) may also be dispersing via gene flow, because there are no distinct patterns in these analyses (Figure 1b, Figure S1c). However, future work should investigate the variants associated with NTSR resistance to obtain a clearer picture of gene flow of this type of resistance. Short‐ and long‐distance gene flow can occur through seed movement facilitated by human activities, as well as over short distances via wind or animal dispersal (Karn and Jasieniuk 2017). It has been found that *B. tectorum* , although predominantly a self‐pollinated species, may exhibit occasional outcrossing events, particularly influenced by environmental conditions, which could explain the level of admixture observed in this study (Adams and Allard 1982; Meyer et al. 2013). In addition to the multiple ALS mutations reported, evidence also suggests that some populations exhibit ALS inhibitor resistance through mechanisms other than target site mutations, indicating that multiple resistance mechanisms are present in the region, likely resulting from additional independent origins of resistance (Table S3).

Our results contribute to a growing body of evidence supporting the repeated evolution of herbicide resistance in agricultural weeds. *B. tectorum* provides a prime example of how plants respond to strong anthropogenic‐driven selection pressure and the multitude of paths that evolution finds to respond to the same selection pressure. The results of this study suggest multiple evolutionary origins of ALS resistance in *B. tectorum* in northeastern Oregon. We found nine *ALS* mutations responsible for resistance at the target site and observed an admixture of resistance alleles within populations. The spread of resistance alleles likely occurs through gene flow via pollen and/or seed dispersal and by multiple instances of resistance mutation evolution within weed populations. We also found evidence of NTSR evolution to ALS‐inhibiting herbicides in *B. tectorum* that warrants further investigation. The presence of the PSII resistance‐endowing Ser‐264‐Gly mutation was detected in two populations. The PSII inhibitor‐resistant populations were also ALS inhibitor‐resistant and carried at least one ALS mutation. Unlike *ALS* gene mutations, which are nuclear inherited, the chloroplast *psbA* gene mutations are maternally inherited, which means the dispersal of PSII herbicide‐resistance alleles is mostly by seeds, limiting spread compared with ALS herbicide‐resistance alleles (Powles and Yu 2010).

## Author Contributions

V.H.V.R., C.M.S., J.G. and C.A.C.G.B. conceived and designed the study. V.H.V.R., J.G. and C.A.C.G.B. collected data. V.H.V.R. and C.A.C.G.B. analysed the Rad‐seq data. All authors assisted with data interpretation and manuscript revisions.

## Disclosure

Benefit‐Sharing Statement: Benefits from this research accrue from the sharing of our data and results on public databases as described above.

## Conflicts of Interest

The authors declare no conflicts of interest.

## Supporting information


**Figure S1.** Principal component analysis (PCA) of *Bromus tectorum* populations. (a) PCA including *B. tectorum* populations and outgroup. (b) PCA with PC1 and PC2. (c) PCA with populations labelled as having target site (TSR) or nontarget site resistance (NTSR), and susceptible individuals. (d) PCA showing mutation Pro‐197‐His.
Figure S2. Cross‐validation plot showing error versus K. The K with the best model fit minimises error values.
Figure S3. Rooted Neighbour‐joining tree including the *B. tectorum* populations and the outgroup ( *B. diandrus* ; WAS6). The blue arrow highlights the base of the clade that includes the ‘blue’ ADMIXTURE population.
Figure S4. Isolation‐by‐distance plot with pairwise genetic and geographic distance between *B. tectorum* individuals. The mantel test was performed with 10,000 simulations (*p* = 0.15).
Table S1. Primers used for ALS gene sequencing.

## Data Availability

Raw sequencing data is available under the NCBI Sequence Read Archive BioProject number PRJNA1170238. Codes used in the analyses are available at https://github.com/caiobrunharo/Bromus_tectorum_Oregon.
