# Supplementary material for: Multiple Origins or Widespread Gene Flow in Agricultural Fields? Regional Population Genomics of Herbicide Resistance in Bromus tectorum
Source: Mol Ecol. 2025 May 8;34(11):e17791. doi: 10.1111/mec.17791 (PMC12100591; doi:10.1111/mec.17791)
Supplement: Supplementary file 1 — Figure S1. Principal component analysis (PCA) of Bromus tectorum populations. (a) PCA including B. tectorum populations and outgroup. (b) PCA with PC1 and PC2. (c) PCA with populations labelled as having target site (TSR) or nontarget site resistance (NTSR), and susceptible individuals. (d) PCA showing mutation Pro‐197‐His. Figure S2. Cross‐validation plot showing error versus K. The K with the best model fit minimises error values. Figure S3. Rooted Neighbour‐joining tree including the B. tectorum populations and the outgroup ( B. diandrus ; WAS6). The blue arrow highlights the base of the clade that includes the ‘blue’ ADMIXTURE population. Figure S4. Isolation‐by‐distance plot with pairwise genetic and geographic distance between B. tectorum individuals. The mantel test was performed with 10,000 simulations (p = 0.15). Table S1. Primers used for ALS gene sequencing. [file MEC-34-e17791-s001.pdf]

MOLECULAR ECOLOGY

Supplemental Information for:

Multiple evolutionary origins or widespread gene flow of herbicide resistance in Bromus tectorum? Insights into genetic variation and population structure

Victor H. V. Ribeiro, Joseph Gallagher, Carol Mallory-Smith, Judit Barroso, and Caio A. C. G. Brunharo

Table of Contents:

**Figure S1**	Page 2
**Figure S2**	Page 5
**Figure S3**	Page 6
**Figure S4**	Page 7
**Table S1**	Page 8
**Table S2**	Page 9
**Table S3**	Page 10

MOLECULAR ECOLOGY

(a)

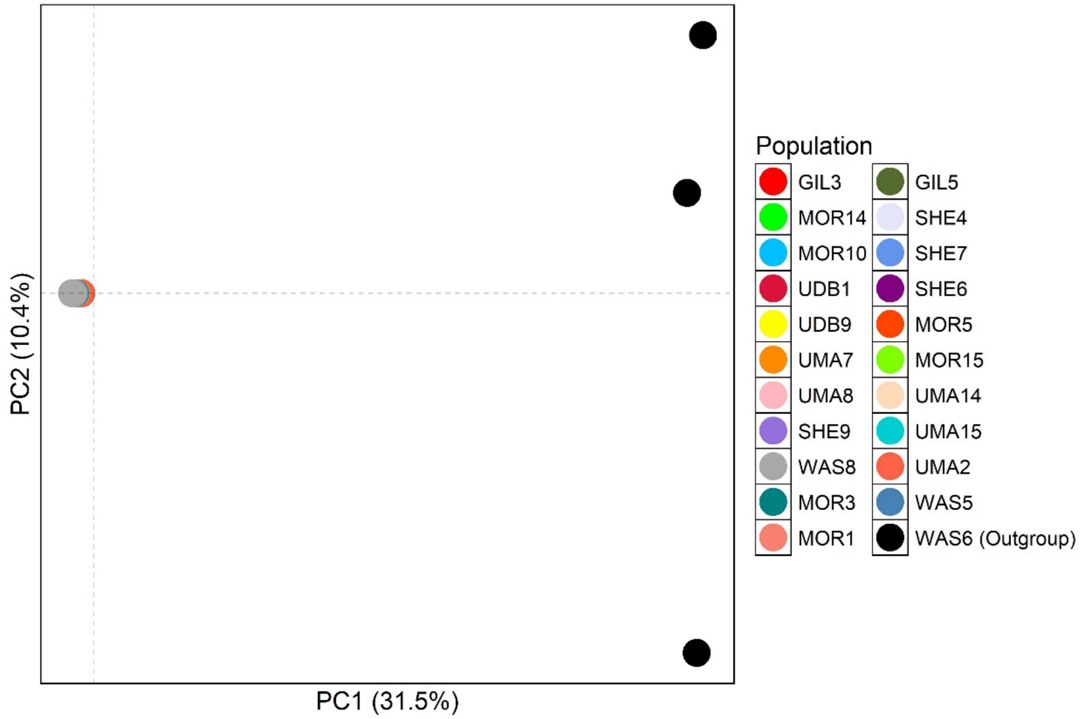

(b)

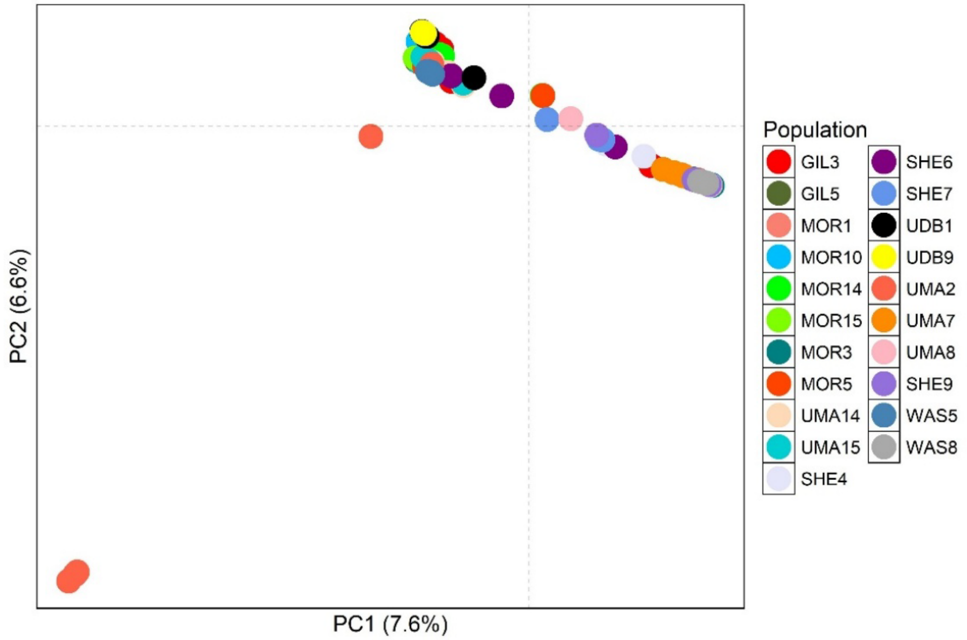

MOLECULAR ECOLOGY

(c)

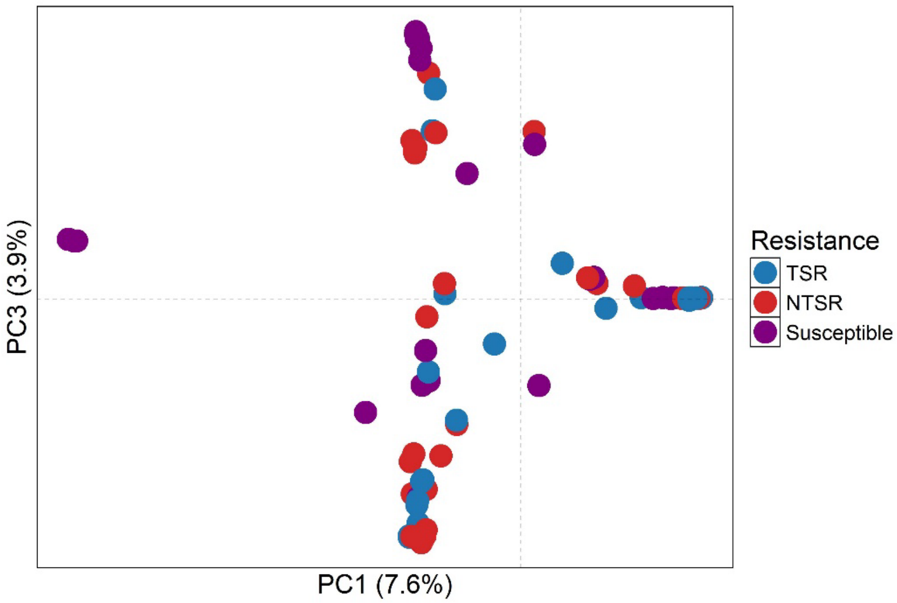

(d)

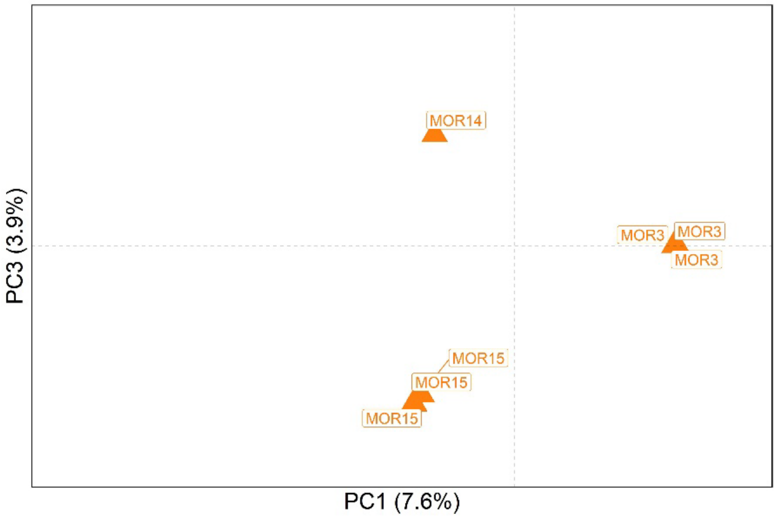

MOLECULAR ECOLOGY

Figure S1. Principal component analysis (PCA) of *Bromus tectorum* populations. (a) including *B. tectorum* populations and outgroup. (b) underscoring the separation of *B. tectorum* populations based on resistant individuals with target site mutations (TSR), resistant individuals without target site mutations (NTSR), and susceptible individuals. (c) PCA with individuals plotted between PC1 and PC2. (d) underscoring the separation of *B. tectorum* populations carrying the Pro-197-His mutation.

MOLECULAR ECOLOGY

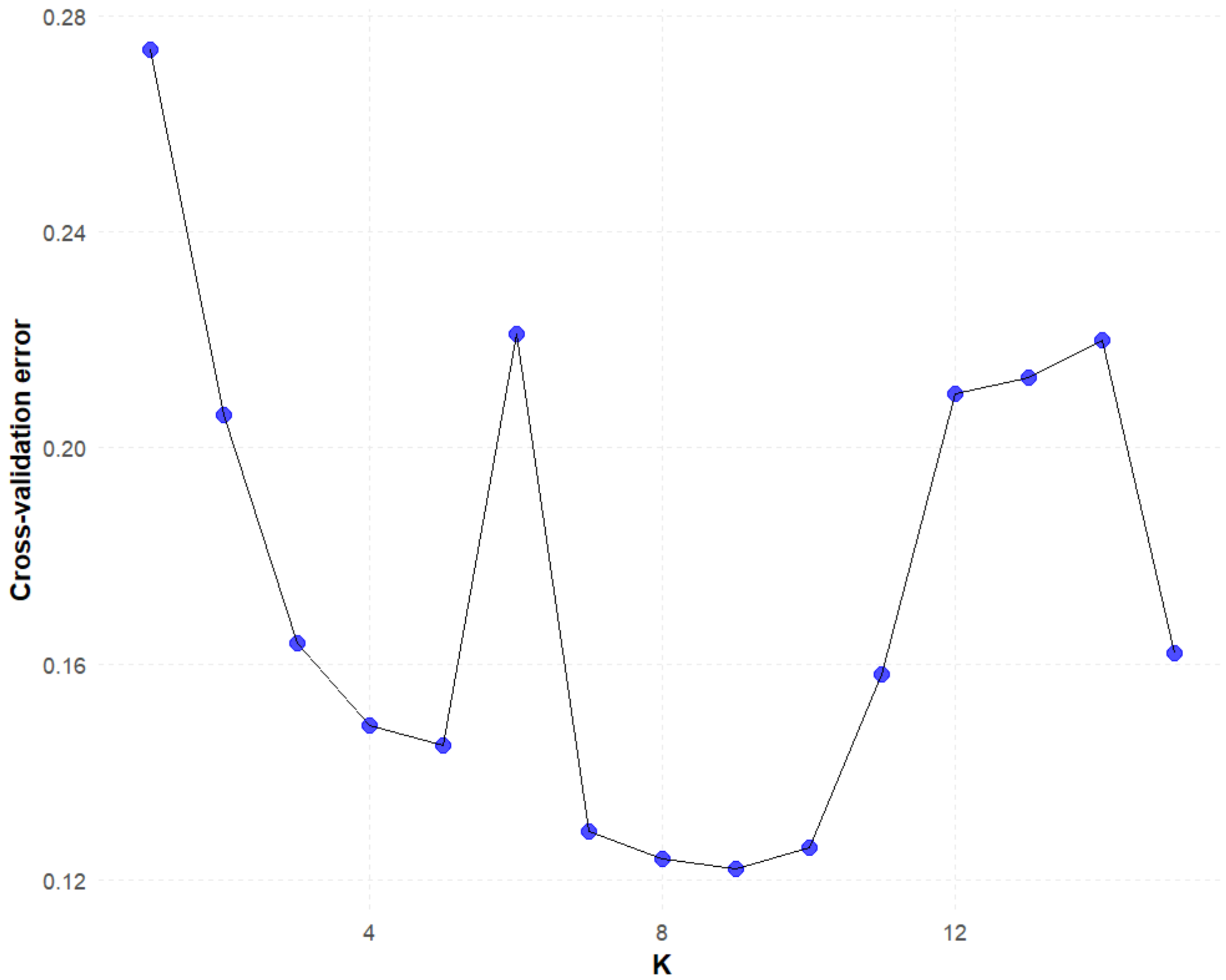

Figure S2. Cross-validation plot showing error versus *K*. The K with the best model fit minimizes error values.

MOLECULAR ECOLOGY

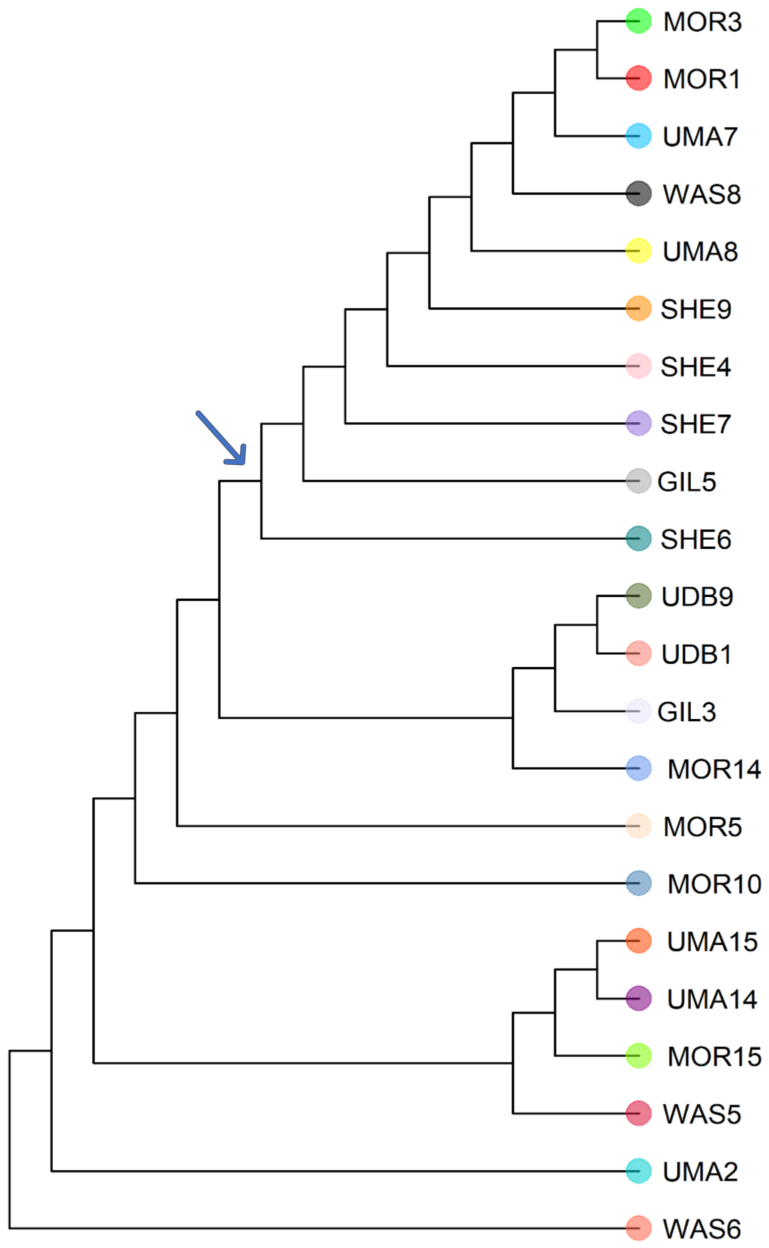

Figure S3. Rooted Neighbor-joining tree including the *B. tectorum* populations and the outgroup (*B. diandrus*; WAS6). The blue arrow highlights the base of the clade that includes the "blue" ADMIXTURE population.

MOLECULAR ECOLOGY

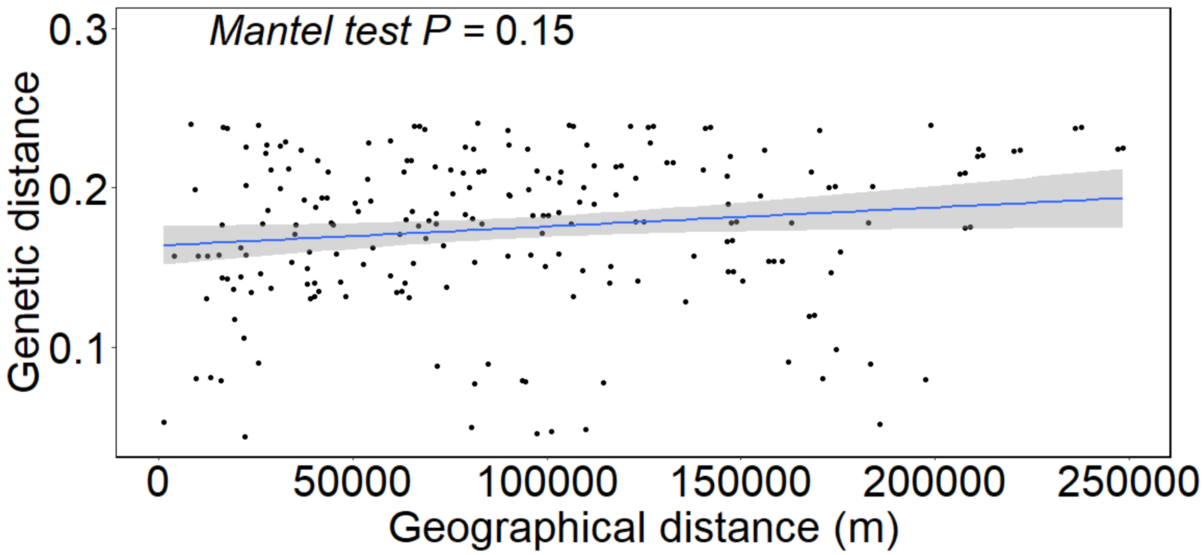

Figure S4. Isolation-by-distance plot with pairwise genetic and geographic distance between *B. tectorum* individuals. The Mantel test was performed with 10,000 simulations ($P = 0.15$).

Table S1. Primers used for *ALS* gene sequencing.

Primers	Sequence (5'-3')	Ta (°C)[d]	Fragment (bp)	Position
Kumar_F[a]	GTCGACGTCTTCGCCTACC	68	1,271	Ala-122, Pro-197, and Ala-205
G_F1[b]	CGACGTCTTCGCCTACCC			
F_770[b]	CACCATCTACTGAATCGCTTGAG	66	762	Asp-376 and Arg-377
R_1533[b]	CATCAATGTCAACAACTGTAACACC			
Park_F[c]	GGGCAGCACCAGATGTGGGCG	62	556	Trp-574, Ser-653, and Gly-654
Kumar_R[a]	AATATTCGATCCTGCCATCA			

[a]Primer sequences previously described by Kumar & Jha (2017).
[b]Primer sequences designed using Primer 3 Plus software.
[c]Primer sequence previously described by Park & Mallory-Smith (2004).
[d]Primer annealing temperature.

MOLECULAR ECOLOGY

Table S2. *Bromus tectorum* populations collected from wheat and fine fescue fields included in the population genomics analysis.

Population	Latitude	Longitude	Sample number
GIL3	45.3879306	-120.1543083	4
GIL5	45.5751889	-120.1952083	4
MOR1	45.35834444	-119.8144778	5
MOR3	45.49412778	-119.6069194	5
MOR5	45.28898	-119.8833783	4
MOR10	45.508159	-120.006801	5
MOR14	45.436833	-119.882821	4
MOR15	45.5599667	-119.6548639	4
SHE4	45.5478167	-120.6940694	4
SHE6	45.4083139	-120.66705	4
SHE7	45.441375	-120.6466444	4
SHE9	45.569144	-120.813525	4
UDB1[1]	45.3138917	-118.015275	5
UDB9[1]	45.3096611	-117.9977138	5
UMA2	45.7457611	-119.3927333	5
UMA7	45.722886	-118.625071	4
UMA8	45.7877222	-118.4781667	4
UMA14	45.95583333	-118.8977778	4
UMA15	45.78555556	-118.8327778	5
WAS5	45.513	-121.163	4
WAS8	45.617	-121.009	5

MOLECULAR ECOLOGY

Table S3. Phenotype and sequence analyses of *B. tectorum* populations collected from wheat fields.[a]

Populations	ALS resistance status[b]	*ALS* mutations (frequency)	PSII resistance status[c]	*psbA* mutation (frequency)
GIL1	**Resistant**	**NTSR (4/4)**	**Susceptible**	**WT**
GIL2	**Resistant**	**NTSR (4/4)**	**Susceptible**	**WT**
GIL3	**Resistant**	**Pro-197-Leu (2/4)/Pro-197-Thr (1/4)**	**Resistant**	**Ser-264-Gly (4/4)**
GIL4	**Resistant**	**NTSR (4/4)**	**Susceptible**	**WT**
GIL5	**Resistant**	**NTSR (4/4)**	**Susceptible**	**WT**
GIL6	**Resistant**	**NTSR (4/4)**	**Susceptible**	**WT**
MOR1	**Resistant**	**Pro-197-Thr (2/4)**	**Susceptible**	**WT**
MOR2	**Resistant**	**Pro-197-Thr (1/4)**	**Susceptible**	**WT**
MOR3	**Resistant**	**Pro-197-Thr (1/4)/Pro-197-His (2/4)**	**Susceptible**	**WT**
MOR4	**Resistant**	**Pro-197-Leu (2/4)/Asp-376-Glu (1/4)**	**Susceptible**	**WT**
MOR5	**Susceptible**	**WT (4/4)**	**Susceptible**	**WT**
MOR6	**Resistant**	**Ser-653-Asn (4/4)**	**Susceptible**	**WT**
MOR7	**Resistant**	**Pro-197-His (1/4)**	**Susceptible**	**WT**
MOR8	**Resistant**	**NTSR (4/4)**	**Susceptible**	**WT**
MOR9	**Resistant**	**NTSR (4/4)**	**Susceptible**	**WT**
MOR10	**Resistant**	**NTSR (4/4)**	**Susceptible**	**WT**
MOR11	**Resistant**	**NTSR (4/4)**	**Susceptible**	**WT**
MOR13	**Resistant**	**NTSR (4/4)**	**Susceptible**	**WT**
MOR14	**Resistant**	**Pro-197-His (1/4)**	**Susceptible**	**WT**
MOR15	**Resistant**	**Pro-197-His (3/4)**	**Susceptible**	**WT**
SHE1	**Resistant**	**NTSR (4/4)**	**Susceptible**	**WT**
SHE2	**Resistant**	**NTSR (4/4)**	**Susceptible**	**WT**
SHE4	**Resistant**	**NTSR (4/4)**	**Susceptible**	**WT**
SHE5	**Resistant**	**Pro-197-Ser (2/4)**	**Susceptible**	**WT**
SHE6	**Resistant**	**Pro-197-Thr (3/4)**	**Resistant**	**Ser-264-Gly (4/4)**
SHE7	**Susceptible**	**WT (3/4)**	**Susceptible**	**WT**
SHE8	**Resistant**	**Pro-197-Leu (1/4)**	**Susceptible**	**WT**
SHE9	**Resistant**	**NTSR (4/4)**	**Susceptible**	**WT**
SHE10	**Susceptible**	**WT (4/4)**	**Susceptible**	**WT**
SHE11	**Resistant**	**NTSR (4/4)**	**Susceptible**	**WT**
SHE12	**Resistant**	**Ala-122-Thr (2/4)**	**Susceptible**	**WT**
UMA1	**Susceptible**	**WT (4/4)**	**Susceptible**	**WT**
UMA2	**Susceptible**	**WT (4/4)**	**Susceptible**	**WT**

UMA3	**Susceptible**	**WT (4/4)**	**Susceptible**	**WT**
UMA4	**Resistant**	**Pro-197-Leu (1/4)/Asp-376-Glu (1/4)/Trp-574-Leu (1/4)**	**Susceptible**	**WT**
UMA5	**Susceptible**	**WT (3/4)**	**Susceptible**	**WT**
UMA6	**Susceptible**	**WT (4/4)**	**Susceptible**	**WT**
UMA7	**Susceptible**	**WT (2/4)**	**Susceptible**	**WT**
UMA8	**Resistant**	**Ala-205-Val (2/4)/Pro-197-Leu (1/4)**	**Susceptible**	**WT**
UMA9	**Resistant**	**Asp-376-Glu (4/4)**	**Susceptible**	**WT**
UMA10	**Resistant**	**NTSR (4/4)**	**Susceptible**	**WT**
UMA11	**Resistant**	**Ala-205-Val (4/4)**	**Susceptible**	**WT**
UMA12	**Resistant**	**Ala-205-Val (2/4)**	**Susceptible**	**WT**
UMA13	**Susceptible**	**WT (4/4)**	**Susceptible**	**WT**
UMA14	**Resistant**	**NTSR (3/4)**	**Susceptible**	**WT**
UMA15	**Resistant**	**Pro-197-Thr (4/4)**	**Susceptible**	**WT**
WAS5	**Resistant**	**NTSR (4/4)**	**Susceptible**	**WT**
WAS7	**Resistant**	**Pro-197-Leu (2/4)**	**Susceptible**	**WT**
WAS8	**Resistant**	**Pro-197-Leu (3/4)**	**Susceptible**	**WT**

[a]Abbreviations: NTSR, non-target-site resistance (ALS-resistant individuals with no detectable mutations); WT, wild type.
[b]Sulfosulfuron was used in the ALS resistance testing.
[c]Metribuzin was used in the PSII resistance testing.
